# Radiographic Evaluation of the Prevalence of External Root Resorption in Patients With Periodontitis in Indore: A Cross-Sectional Study

**DOI:** 10.7759/cureus.74402

**Published:** 2024-11-25

**Authors:** Parul Jain, Veena Kalburgi, Ashish Kumar Jain, Madhvika Patidar, Prerna R Batham

**Affiliations:** 1 Department of Periodontology, Government College of Dentistry, Indore, IND; 2 Department of Periodontology and Implantology, People’s College of Dental Sciences, Bhopal, IND; 3 Department of Cardiology, Mahatma Gandhi Memorial Medical College, Indore, IND; 4 Department of Oral Pathology and Microbiology, Government College of Dentistry, Indore, IND; 5 Department of Orthodontics and Dentofacial Orthopedics, Government College of Dentistry, Indore, IND

**Keywords:** external root resorption, generalized periodontitis, localized periodontitis, orthopantomograph, stages of periodontitis

## Abstract

Objective

Permanent teeth roots undergo resorption under pathologic conditions such as trauma, orthodontic treatment, pulpal infections, periodontitis, and periodontal therapy. The present study aimed to determine the prevalence of external root resorption (ERR) in patients with periodontitis as seen in orthopantomography (OPGs).

Methodology

This single-center, retrospective, cross-sectional radiographic study was conducted from January 2021 to December 2022, including 656 orthopantomographs (OPGs) from patients with periodontitis. OPGs were evaluated based on specific inclusion and exclusion criteria. ERR was assessed across mild, moderate, and severe periodontitis cases (Stage I/II/III/IV according to the 2017 American Academy of Periodontology) in both localized and generalized periodontitis and in cases of horizontal and vertical bone loss/defects. Data analysis was performed using IBM SPSS Statistics for Windows, Version 25 (Released 2017; IBM Corp., Armonk, New York, United States). Descriptive statistics were calculated and presented as numbers, percentages, and mean ± standard deviation. Categorical variables were compared using the chi-square test, with a p-value < 0.05 considered statistically significant.

Results

The study analyzed 656 subjects, with 226 (34.5%) showing external root resorption (ERR). ERR prevalence was higher among males (122, 38.6%) compared to females (104, 30.6%) (p = 0.031). ERR was more common in subjects with vertical bone loss (105, 49.1%) than those without (121, 27.4%) (p < 0.001) and in those with horizontal bone defects (55, 45.8%) compared to those without (171, 31.9%) (p = 0.004). By periodontitis severity, ERR occurred in Stage I (61, 21.2%), Stage II (108, 37.4%), and Stage III/IV (57, 72.2%) (p = 0.001). Localized periodontitis showed higher ERR prevalence (40, 44.0%) than generalized periodontitis (186, 32.9%) (p = 0.040). Age was not significantly associated with ERR (p = 0.423).

Conclusion

ERR was significantly associated with the severity of periodontitis, localized and generalized periodontitis, and horizontal and vertical bone defects. Prompt diagnosis and treatment planning are essential for preserving teeth affected by ERR, thereby supporting masticatory function, aesthetics, self-esteem, and the overall oral health-related quality of life for patients.

## Introduction

Periodontitis is a chronic, multifactorial immune-inflammatory disease that affects the periodontal structures, primarily caused by the dysbiosis of microorganisms [1,2]. The clinical features of periodontitis include bleeding on probing, periodontal pockets, loss of attachment, gingival recession, halitosis, mobility, pathological migration, root resorption, and tooth loss [3,4]. Root resorption, as defined by Ne et al., is “a condition associated with either a physiologic or a pathologic process resulting in the loss of dentin, cementum, or bone” [5]. Typically, the dental hard tissues of permanent teeth, namely dentin, cementum, and enamel, do not undergo resorption. However, resorption may occur in permanent teeth in response to pathological conditions such as trauma, chronic inflammation of the pulp and periodontal tissues, orthodontic forces exerting pressure on the periodontal ligament, or conditions like cysts and tumors [6,7].

Radiographs play a vital role in diagnosing periodontal disease, reflecting changes in hard tissues [8]. The diagnosis of external root resorption (ERR) can be achieved through various imaging modalities, including orthopantomography (OPG), intraoral periapical radiographs (IOPA), cone beam computed tomography (CBCT), histologic examinations, and surgical exposure. Untreated or inadequately treated periodontitis may lead to dental mobility and eventual tooth loss, resulting in edentulism [9]. This condition negatively impacts oral health by causing aesthetic issues, disability, and dysfunction of the masticatory system, in addition to imposing significant psychosocial and economic burdens [10,11].

In the current era of precision medicine, which focuses on identifying the most effective treatment approaches based on genetic, environmental, and lifestyle factors [12], it is crucial to ensure appropriate and prompt treatment of periodontitis and ERR to maximize the preservation of tooth and periodontal structures. Thus, it is essential to determine the prevalence of ERR in patients with periodontitis within the general population. This study will direct researchers' and clinicians’ attention to the pathological processes involved and facilitate appropriate therapeutic interventions to improve prognosis. This study addressed the gaps in the existing literature regarding the prevalence of ERR among patients with periodontitis (Stages I, II, III, and IV) in the Indore district population. Specifically, this study aimed to radiographically assess the prevalence of ERR across various stages of periodontitis, evaluate the percentage of teeth exhibiting ERR in both localized and generalized forms of periodontitis, and investigate the percentage of teeth demonstrating ERR associated with horizontal and vertical bone loss or defects.

## Materials and methods

Study design and setting

This retrospective, cross-sectional study aimed to assess the prevalence and characteristics of external root resorption (ERR) among patients with varying stages of periodontitis. The study was conducted within the Department of Periodontology and Implantology at the Government College of Dentistry, Indore. The study received approval from the Institutional Review Board (IRB Approval No.: 01 PU/DAA/Ph.D./Registration/11). The research utilized orthopantomogram (OPG) records, specifically analyzing radiographic images of patients who visited the department for periodontal evaluations between January 2021 and December 2022.

Population, sample size, and sampling technique

The study population included patients who met predefined eligibility criteria within the specified timeframe. A sample size of approximately 650 patients was calculated to ensure adequate statistical power, with an assumed margin of error of 5% at a 95% confidence interval. Purposive sampling was employed to select cases that adhered to the inclusion and exclusion criteria, facilitating a focused analysis of ERR within this cohort. 

Inclusion and exclusion criteria

The inclusion criteria required that patients be between 18 and 65 years old, have at least 14 natural teeth (defined as original, permanent teeth that a patient has), and provide high-quality, standardized orthopantomograms (OPGs) taken with a single imaging device. Patients were excluded if their OPGs had poor image quality, had cavities with periapical involvement, exhibited significant wear on their teeth, were receiving orthodontic treatment, or showed signs of cysts or tumors on the X-rays.

Data collection and radiographic assessment

Data collection involved a systematic review of OPG records to gather demographic information, including age and gender, as well as clinical details relevant to periodontal assessment. The OPG images were analyzed to evaluate the presence and extent of external root resorption (ERR), with trained dental professionals performing the radiographic assessments to ensure reliability.

The stages of periodontitis were categorized into four levels: Stage I represented initial periodontitis, characterized by mild inflammation with pocket depths of 4-5 mm and localized attachment loss. Stage II was defined as moderate periodontitis, with pocket depths of 5-6 mm and moderate inflammation accompanied by increased attachment loss. Stage III, or severe periodontitis, marked by pocket depths greater than 6 mm, significant attachment loss, and the potential for tooth mobility, indicated a risk of tooth loss. Finally, Stage IV represented advanced periodontitis, involving extensive attachment loss and considerable tooth mobility, highlighting a significant risk for tooth loss.

Assessment of external root resorption (ERR)

ERR was confirmed based on the criteria established by Al-Khateeb and Bataineh [13]. The assessment involved careful examination of the OPG images to identify ERR and categorize it based on its location within the root structure into cervical, middle, or apical thirds. Severity was graded according to the criteria proposed by Ericson et al. [14], with ERR classified as slight (involving minimal dentin), moderate (approximately half of the dentin thickness), or severe (involving the pulp cavity). Additionally, ERR was categorized by directionality, with horizontal ERR indicating resorption along the horizontal plane of the root and vertical ERR indicating resorption along the vertical axis.

Data analysis

Statistical analysis was performed using IBM SPSS Statistics for Windows, Version 25 (Released 2017; IBM Corp., Armonk, New York, United States). Descriptive statistics summarized demographic and clinical characteristics, which were presented as frequencies, percentages, and means with standard deviations. Inferential analysis was conducted using the chi-square test to explore associations between categorical variables, specifically investigating the relationship between ERR and the stages of periodontitis. A significance level of p < 0.05 was established for all statistical tests.

Quality assurance

To ensure data reliability and minimize observer bias, all OPG assessments were conducted by trained dental professionals who underwent calibration exercises prior to the study. Each OPG was independently reviewed twice, and any discrepancies were addressed in consultation with a senior periodontist. This approach ensured consistency and accuracy in identifying ERR and evaluating periodontal conditions. 

## Results

The present study analyzed the OPGs of 656 subjects, revealing a 226 (34.5%) prevalence of external root resorption (ERR) among periodontitis patients. Of these, 111 (16.9%) showed ERR in a single tooth, while 115 (17.5%) had ERR in multiple teeth. Among those with ERR, 28 (4.3%) had it in maxillary teeth only, 136 (20.7%) in mandibular teeth only, and 62 (9.5%) in both arches. The mean age of the subjects was 45.2 ± 8.396 years, with no significant association between age and the occurrence of ERR. In the youngest age group, less than 30 years old, seven (31.8%) subjects exhibited ERR, while 15 (68.2%) did not. Among those aged 31-40 years, 52 (33.1%) subjects had ERR, compared to 105 (66.9%) who did not. The 41-50 years age group, which represents the largest group with 334 individuals, showed a slightly higher prevalence of ERR at 122 (36.5%), with 212 (63.5%) displaying no ERR. In the 51-60 year group, 33 (28.4%) subjects demonstrated ERR, while the majority, 83 (71.6%), did not. Lastly, in the 61-70 year age group, ERR was observed in 12 (44.4%) subjects, with 15 (55.6%) showing no ERR. Overall, 226 (34.5%) of the total population presented with ERR, while 430 (65.5%) did not. A chi-square test for independence revealed no significant association between age and the occurrence of ERR (χ² = 3.873, df = 4, p = 0.423), indicating that the prevalence of ERR was not significantly affected by age within the study population (Table 1).

**Table 1:** Association between ERR and age ERR: external root resorption Data is presented in N (%) format

Age group	External root resorption		Total	Chi-square value	df	p-value
	Yes	No				
Less than 30 years	7 (31.8%)	15 (68.2%)	22 (100%)	3.873	4	0.423
31-40 years	52 (33.1%)	105 (66.9%)	157 (100%)			
41-50 years	122 (36.5%)	212 (63.5%)	334 (100%)			
51-60 years	33 (28.4%)	83 (71.6%)	116 (100%)			
61-70 years	12 (44.4%)	15 (55.6%)	27 (100%)			
Total	226 (34.5%)	430 (65.5%)	656 (100%)			

Among the 316 male subjects, 122 (38.6%) had ERR, while 194 (61.4%) did not. In comparison, out of 340 female subjects, 104 (30.6%) exhibited ERR, and 236 (69.4%) did not. Overall, 226 (34.5%) of the total 656 subjects showed ERR, while 430 (65.5%) showed no ERR. The chi-square test showed a significant association between gender and the presence of ERR, with a chi-square value of 4.664, 1 degree of freedom, and a p-value of 0.031 (*p < 0.05), indicating a higher prevalence of ERR among males compared to females (Table 2).

**Table 2:** Association between ERR and gender *: indicates significant p-value; ERR: external root resorption Data is presented in N (%) format

Gender	External root resorption		Chi-square value	df	p-value
	Yes	No			
Male	122 (38.6%)	194 (61.4%)	4.664	1	0.031*
Female	104 (30.6%)	236 (69.4%)			
Total	226 (34.5%)	430 (65.5%)			

Among the 214 subjects with vertical bone loss, 105 (49.1%) exhibited ERR, while 109 (50.9%) did not. In contrast, among the 442 subjects without vertical bone loss, 121 (27.4%) had ERR, and 321 (72.6%) did not. Overall, 226 (34.5%) of the total 656 subjects revealed ERR, while 430 (65.5%) showed no ERR. The chi-square test indicated a highly significant association between the presence of vertical bone loss and ERR, with a chi-square value of 30.038, 1 degree of freedom, and a p-value of <0.001 (*p < 0.05), suggesting that ERR was more prevalent in subjects with vertical bone loss compared to those without (Table 3).

**Table 3:** Association between ERR and vertical bone defect *: indicates significant p-value; ERR: external root resorption Data is presented in N (%) format

Vertical bone defect	External root resorption		Total N (%)	Chi-square value	df	p-value
	Yes	No				
Yes	105 (49.1%)	109 (50.9%)	214	30.038	1	<0.001*
No	121 (27.4%)	321 (72.6%)	442			
Total	226 (34.5%)	430 (65.5%)	656			

Among the 536 subjects with horizontal bone defects, 171 (31.9%) exhibited ERR, while 365 (68.1%) did not. In contrast, among the 120 subjects without horizontal bone defects, 55 (45.8%) showed ERR and 65 (54.2%) did not. Overall, ERR was identified in 226 (34.5%) of the total 656 subjects, while 430 (65.5%) showed no ERR. The chi-square test revealed a significant association between horizontal bone defects and ERR, with a chi-square value of 8.426, 1 degree of freedom, and a p-value of 0.004 (*p < 0.05), indicating that ERR was more prevalent in subjects with horizontal bone defects compared to those without (Table 4).

**Table 4:** Association between ERR and horizontal bone defect *: indicates significant p-value; ERR: external root resorption Data is presented in N (%) format

Horizontal bone defect	External root resorption		Chi-square	p-value
	Yes	No		
Yes	171 (31.9%)	365 (68.1%)	8.426	0.004*
No	55 (45.8%)	65 (54.2%)		
Total	226 (34.5%)	430 (65.5%)		

Among subjects with mild periodontitis (Stage I), 61 (21.2%) had ERR, while 227 (78.8%) did not, out of a total of 288 subjects. In the moderate periodontitis group (Stage II), 108 (37.4%) had ERR and 181 (62.6%) did not, for a total of 289 subjects. For subjects with severe periodontitis (Stage III/IV), 57 (72.2%) exhibited ERR, while 22 (27.8%) did not, from a total of 79 subjects. Overall, 226 (34.5%) of the 656 subjects showed ERR, while 430 (65.5%) showed no ERR. The chi-square test revealed a significant association between the severity of periodontitis and the occurrence of ERR, with a chi-square value of 73.273, 2 degrees of freedom, and a p-value of 0.001 (*p < 0.05), indicating that ERR prevalence increased with greater severity of periodontitis (Table 5).

**Table 5:** Association between ERR and severity of periodontitis *: indicates significant p-value; ERR: external root resorption Data is presented in N (%) format

Severity of periodontitis	External root resorption		Total	Chi-square value	df	p-value
	Yes	No				
Mild	61 (21.2%)	227 (78.8%)	288 (100%)	73.273	2	0.001*
Moderate	108 (37.4%)	181 (62.6%)	289 (100%)			
Severe	57 (72.2%)	22 (27.8%)	79 (100%)			
Total	226	430 (65.5%)	656 (100%)			

Among the 91 subjects with localized periodontitis, 40 (44.0%) exhibited ERR, while 51 (56.0%) did not. In contrast, of the 565 subjects with generalized periodontitis, 186 (32.9%) had ERR, and 379 (67.1%) did not. Overall, 226 (34.5%) of the 656 total subjects had ERR, while 430 (65.5%) showed no ERR. The chi-square test indicated a significant association between the type of periodontitis (localized or generalized) and the presence of ERR, with a chi-square value of 4.227, 1 degree of freedom, and a p-value of 0.040 (*p < 0.05), suggesting a higher prevalence of ERR in subjects with localized periodontitis (Table 6).

**Table 6:** Association between ERR and extent of periodontitis *: indicates significant p-value; ERR: external root resorption Data is presented in N (%) format

Severity of periodontitis	External root resorption		Total N (%)	Chi-square value	df	p-value
	Yes	No				
Localized	40 (44%)	51 (56%)	91 (100%)	4.227	1	0.040*
Generalized	186 (32.9%)	379 (67.1%)	565 (100%)			
Total	226 (34.5%)	430 (65.5%)	656 (100%)			

## Discussion

This retrospective study utilized a radiographic approach, including 656 orthopantomograms (OPGs), to assess the prevalence of external root resorption (ERR) among individuals with periodontitis in the mixed urban and rural population of Indore district. A total of 2852 OPGs, originally recommended by the Department of Periodontics for various periodontal pathologies, were reviewed. Among these, 656 OPGs demonstrated periodontitis. OPG was selected as it is one of the most commonly used diagnostic imaging methods in periodontics. Each eligible OPG was analyzed for signs of ERR in cases of mild, moderate, and severe periodontitis, as well as in both localized and generalized periodontitis and horizontal and vertical bone loss or defects.

In this study, no statistically significant relationship was found between age and ERR. In research by Henry and Weinmann, it was reported that in patients aged 16 to 58 years, 90.5% of teeth showed root resorption [15]. Another study demonstrated that 77% of teeth in patients over 50 years with adult periodontitis (AP) had root resorption [16]. Crespo et al. further observed that in patients aged 43-80 years, 98.5% of teeth affected by AP exhibited resorption [17].

The present study identified mandibular first molars as the teeth most commonly affected by ERR, with a prevalence of 29.7%, likely due to furcation involvement and difficulties in maintaining oral hygiene [18]. This finding contrasts with earlier studies, which reported mandibular incisors as the most affected, with 84.61% displaying root resorption [7]. Similar findings were observed by Bossert and Marks [19] and Crespo et al. [17], who suggested that mandibular incisors may be more susceptible to root resorption due to their slender anatomy, limited surrounding periodontal structures, and higher vulnerability to periodontal destruction.

The severity of periodontitis in this study was significantly associated with ERR, with 21.2%, 37.4%, and 72.2% of teeth affected in cases of mild, moderate, and severe periodontitis, respectively. Mahajan et al. reported a range of 13.33% to 93.33% resorption linked to periodontal disease, with resorption frequency increasing alongside disease severity [7]. Comparable results were found in studies by López et al. [16], Harvey and Zander [20], and Crespo et al., who observed 100% resorption in severely affected teeth [17]. Rodriguez-Pato et al. also reported resorption rates of 46.67%, 85%, and 93.55% in cases of slight, moderate, and severe periodontitis, respectively, with a threefold higher occurrence of resorption in affected teeth [21,22].

Previous studies indicate that the location of resorption varies with marginal tissue level and pocket depth, with resorption most commonly in the apical third of the root, followed by the middle and gingival thirds [7,21,23]. This pattern is thought to occur due to the organic composition and lesser mineralization of cellular cementum in the apical region [24]. Some studies have also noted greater depth and length of resorption in multirooted molars, particularly on the mesial surface [7,21,25-27]. However, as this study relied on radiographic OPGs, no histological sectioning or in-depth evaluation of resorption size, length, or volume was conducted.

Variations in findings across studies could be attributed to differences in criteria for periodontal disease classification, radiographic bone destruction assessments, resorption measurement techniques, and sample preparation methods. Mahajan et al. manually measured resorption and used scanning electron microscopy (SEM) for observation, whereas Rodriguez-Pato et al. employed light microscopy [7,21].

Severe periodontitis is frequently associated with excessive occlusal trauma, which further aggravates resorption. As resorption areas expand in periodontally compromised teeth, occlusal forces exerted on mobile teeth act more laterally, potentially explaining resorption in locations beyond the apical third. Trauma in the cervical third, where cementum overlaps enamel or anatomical gaps exist, can increase resorption susceptibility [21,28]. 

This study also found a significant association between ERR and localized and generalized periodontitis, with a notably higher frequency of ERR in localized cases. A significant association was observed between ERR and vertical and horizontal bone defects. However, to the best of our knowledge, there is limited literature examining the relationship between ERR and the classification of periodontitis as localized or generalized, as well as with vertical and horizontal bone defects.

Globally, periodontitis is a highly prevalent and severe disease, with numerous barriers impeding access to adequate oral health treatment. Behavioral risk factors, socioeconomic constraints, limited access to dental care, and low public awareness contribute to treatment delays, exacerbating functional and aesthetic impacts [4]. Oral health-related quality of life (OHRQoL) reflects how oral health affects social duties, daily functioning, and behavior [27]. Research indicates a strong association between periodontitis severity and reduced OHRQoL, underscoring the need for effective prevention and treatment strategies to mitigate the impact of chronic periodontitis on individuals’ lives [29,30].

Limitations

This investigation had several limitations. The study was retrospective, cross-sectional, and conducted at a single center with a predominantly low socioeconomic patient base. Clinical assessments of bleeding on probing, probing pocket depth, clinical attachment loss, recession, and mobility were not performed. Information on systemic health, risk factors, and specific conditions such as smoking, diabetes, malocclusion, plaque and calculus levels, nutritional deficiencies, and hormonal analyses were also not evaluated. Microbial analysis of plaque or saliva samples, as well as microscopic or histological examination of extracted teeth, were not performed. This study relied solely on OPG imaging; CBCT was not conducted. Since OPGs are two-dimensional, they may not fully capture ongoing root resorption in teeth.

## Conclusions

This study demonstrated a significant association between the severity of periodontitis and the prevalence of external root resorption (ERR). With increasing periodontitis severity, from mild to moderate to severe stages (I to IV), the occurrence of ERR was notably higher. ERR was strongly linked with both localized and generalized forms of periodontitis, as well as with vertical and horizontal bone defects, with molars identified as the most commonly affected teeth. These findings provide valuable insights into the impact of ERR in periodontitis, supporting more accurate diagnosis and treatment planning to prevent disease progression and tooth loss. The results also highlight the importance of timely intervention to preserve self-esteem, aesthetics, and functional efficiency in mastication for individuals affected by periodontitis.
